# Physical function as a predictor of chemotherapy-induced peripheral neuropathy in patients with pancreatic cancer

**DOI:** 10.1186/s12876-024-03227-6

**Published:** 2024-05-06

**Authors:** Kuan-Yin Lin, Po See Chen, Cheng-Feng Lin

**Affiliations:** 1 Department of Physical Therapy, College of Medicine, National Cheng Kung University, No.1, University Road, 701 Tainan, Taiwan; 2 Physical Therapy Center, National Cheng Kung University Hospital, Tainan, Taiwan; 3 School and Graduate Institute of Physical Therapy, National Taiwan University, Taipei, Taiwan; 4 Institute of Behavioral Medicine, College of Medicine, National Cheng Kung University, Tainan, Taiwan

**Keywords:** Chemotherapy, Peripheral neuropathy, Pancreatic cancer, Time Up and go test, Balance

## Abstract

**Background:**

A growing body of research indicates that poor functional status before chemotherapy may be correlated with the severity of chemotherapy-induced peripheral neuropathy (CIPN) after the neurotoxic treatment. However, little is known about the associations between pre-chemotherapy physical function and CIPN in patients with pancreatic cancer.

**Purpose:**

To identify the predictors of CIPN in relation to pre-chemotherapy physical function in patients with pancreatic cancer.

**Methods:**

This secondary analysis included data from patients with pancreatic cancer who participated in a longitudinal research study at National Cheng Kung University Hospital, Tainan, Taiwan. Four physical function tests (i.e., grip strength, Timed Up and Go (TUG), 2-minute step test (2MST), and Romberg test) and two questionnaires (The European Organisation for Research and Treatment of Cancer Quality of Life Questionnaire Core 30 [EORTC QLQ-C30] and Chemotherapy-Induced Peripheral Neuropathy Module [CIPN20]) were assessed at baseline (i.e., before first chemotherapy session) and 2-, 3-, 4-, and 6-month follow-up. Multiple linear regression with adjustment for confounding factors was used to assess the associations between the four functional tests at baseline and the CIPN20 total score and individual subscale scores (sensory, motor, and autonomic) at 6-month follow-up.

**Results:**

Data from a total of 209 pancreatic cancer patients (mean age: 64.4 years, 54.5% male) were analyzed. The findings showed that the severity of CIPN at 6-month follow-up was significantly associated with the baseline TUG completion time (β = 0.684, *p* = 0.003). The TUG completion time was also positively correlated with the 6-month CIPN sensory and autonomic subscales. In addition, a baseline positive Romberg test (β = 0.525, *p* = 0.009) was a significant predictor of the severity of motor neuropathy at 6-month follow-up.

**Conclusion:**

The TUG completion time and positive Romberg test before chemotherapy may be predictive factors of the CIPN severity 6 months after the commencement of chemotherapy. Accordingly, the incorporation of TUG and Romberg tests into the clinical assessment protocol emerges as imperative for individuals diagnosed with pancreatic carcinoma undergoing chemotherapy regimens.

## Introduction

Pancreatic cancer is one of the most common cancers worldwide, with a poor 5-year survival rate of just 6% and a rank of seven among the leading causes of cancer death worldwide [1, 2]. However, a combination of surgery and adjuvant chemotherapy has been shown to improve survival rates for pancreatic cancer compared with those for surgery alone (5-year survival rate of 20.7% vs. 10.4%) [2]. Common chemotherapy drugs after radical resection for pancreatic cancer include modified leucovorin, 5-fluorouracil, irinotecan, oxaliplatin (mFOLFIRINOX), gemcitabine, and capecitabine [3]. While the administration of chemotherapy may improve survival outcomes, it has several undesirable side effects, including hematologic toxicities (e.g., pancytopenia, neutropenia, anemia, and thrombocytopenia), fatigue, and peripheral neuropathy [4].

Peripheral neuropathy is a common side effect of oxaliplatin and nab-paclitaxel, with a prevalence of 19 ∼ 85% [4, 5]. Many factors have been identified as risk factors for chemotherapy-induced peripheral neuropathy (CIPN), including older age, obesity, hypomagnesemia, hypoalbuminemia, anemia, alcohol, diabetes mellitus, inherited neuropathy, endocrinologic and metabolic alterations, medications (such as insulin, metronidazole, misonidazole, sulfasalazine, or phenytoin), and a higher cumulative dose of chemotherapy [6]. Furthermore, a previous study also reported that age and the number of chemotherapy cycles are significant CIPN risk factors in patients with pancreatic cancer [9]. CIPN is generally diagnosed when a patient who is receiving chemotherapy experiences new pain or numbness in the distal portions of the extremities. The diagnosis can be supported by neurological physical examination [7], nerve conduction studies, electromyography, quantitative sensory testing, patient complaints, and general quality of life assessment scales, such as the European Organization for Research and Treatment of Cancer Quality of Life Questionnaire-Chemotherapy-Induced Peripheral Neuropathy 20-item scale (EORTC QLQ-CIPN20) [8].

CIPN is consistently associated with lower self-reported physical function and quality of life during or after chemotherapy [10]. Patients with severe CIPN typically show more significant deterioration in most motor-performance parameters than patients with mild CIPN after adjusting for age [11]. Moreover, balance tests [12], chair stand times, walking speeds, and lower extremity function are all closely associated with the severity of the CIPN symptoms [13]. However, while CIPN and motor performance deficits have been demonstrated in children and adolescents treated for non-central nervous system cancer [12], no study has been undertaken to investigate the relationship between the severity of CIPN and the baseline physical function of patients with pancreatic cancer. Since there are presently no validated effective treatments for CIPN [14], it is important to identify predictive factors for CIPN which could be used to plan tailored therapeutic strategies for patients scheduled for chemotherapy to ensure them a better quality of life [15]. The aim of this study was thus to identify the predictors of CIPN in relation to pre-chemotherapy physical function in patients with pancreatic cancer.

## Methods

This was a secondary analysis of an ongoing longitudinal study aimed at implementing precision medicine for protecting the brain, heart, and activity levels in gastrointestinal malignancies. The study was approved by the Institutional Review Board of National Cheng Kung University Hospital (--/B-ER-110-060).

The inclusion criteria of the longitudinal study were specified as a patient age of ≥ 20 years, a diagnosis of gastrointestinal cancer (stage I-IV), and an Eastern Cooperative Oncology Group performance status (ECOG-PS) score of ≤ 2. In this study, only the data of patients with pancreatic cancer were included in the analysis. The participants were recruited from a university hospital (medical center) from August 2021 until September 2022. All the eligible participants provided written informed consent before enrollment and baseline assessment (i.e., before their first chemotherapy session).

The participants were asked to provide their demographic and family history data, while their medical information was obtained directly from the hospital records. In the original longitudinal study, the participants completed a set of questionnaires regarding the quality of life (European Organization for Research and Treatment of Cancer Quality of Life Core Questionnaire [EORTC QLQ-C30]) [16], EORTC QLQ-CIPN20 [17]). However, only the data of EORTC QLQ-CIPN20 were used in this secondary analysis study as the severity of CIPN symptoms was the main outcome of interest in this study.

The EORTC QLQ-CIPN20 is a 20-item questionnaire evaluating the sensory (9 items), motor (8 items), and autonomic (3 items) symptoms of the patient during the past week, with each item measured on a 4-point Likert scale (1 = “not at all”, 4 = “very much”) [18]. All the scores are converted to a 0–100 scale, with higher scores indicating a greater symptom burden [19]. The EORTC QLQ-CIPN20 questionnaire has strong convergent validity [20] and high reliability [21] in measuring CIPN symptoms. It was thus deemed to be a suitable tool for evaluating the CIPN severity in the present study.

Anthropometric measures (i.e., body mass index) and physical function were also collected. The measures of physical function included grip strength (Jamar dynamometer), mobility, balance, walking ability, fall risk (Timed Up and Go [TUG] test), aerobic capacity (2-minute step test [2MST]), and proprioception (Romberg test). The grip strength was assessed with the elbow of the dominant hand fully extended. The participants were instructed to squeeze the handle of the Jamar dynamometer maximally for three seconds. Three successive measurements (in kilograms) were taken, and the maximum value was used in the analysis [22]. The TUG test was used to assess the physical mobility and balance of the participants by measuring the time (in seconds) taken to stand up from an armchair, walk at a comfortable speed to a line 3 m distant, turn around at the line, walk back to the chair, and sit back down [23]. The aerobic capacity was evaluated by asking the participant to step in place, raising each knee to a height midway between the patella and the iliac crest when standing, for as many times as possible in 2 min. The number of times for which the right knee reached the required height during the observation time was then recorded for analysis purposes [24]. The Romberg test is one of the most commonly used balance (proprioception) tests in cancer populations [25] and is assigned a positive outcome if the participant exhibits increased sway or cannot maintain an upright stance with the feet together (shoes removed) and eyes closed for 30 s [26]. All the tests were performed both before chemotherapy and at 2-, 3, 4, and 6-month follow-up after the first chemotherapy session.

### Analysis

All the analyses were conducted using SPSS Statistics for Windows, Version 17.0. (Chicago: SPSS Inc). Descriptive statistics were used to summarize the demographic and clinical characteristics of the participants. A Kolmogorov-Smirnov test was used to assess normality. Changes in the physical function and CIPN scores over time were analyzed via repeated measures analysis of variance (ANOVA). A Cochran’s Q test was used to evaluate changes in the total number of positive Romberg tests over time. Multiple linear regression with adjustment for confounding factors (i.e., age, body mass index, cancer stage, sex, comorbidities, and baseline CIPN total and subscale scores) was used to assess the associations between the results of the four physical function tests at baseline and the EORTC QLQ-CIPN20 total score and subscale (sensory, motor, and autonomic) scores at 6-month follow-up. A *p*-value of < 0.05 was considered to indicate statistical significance in every case.

## Results

### Demographic and clinical characteristics

The data of a total of 209 participants with pancreatic cancer were included in the analysis. The demographic and clinical characteristics of the participants and the type of chemotherapy regimens used are shown in Table 1. The mean age of the participants was 64.4 (11.0) years, with a range of 30 to 87 years at baseline assessment, and 54.5% were male. The mean body mass index (BMI) was 24.3 ± 16.6 kg/m^2^. The majority (54.4%) of the participants had a cancer stage of IV and the total number of chemotherapy sessions received by each participant was 7.3 ± 6.0. In terms of comorbidities, 41% had diabetes mellitus, 42% had hypertension, and 7% had coronary artery disease. All participants were receiving first-line chemotherapy while enrolled in the study. The most common chemotherapy regimens that participants received were 5-fluorouracil (35.9%), gemcitabine (Gemmis®) (32.1%), and nab-paclitaxel (Abraxane®) (11.5%). Approximately 66% (137/209) of participants were still receiving chemotherapy at the 6-month time point.

**Table 1:** Demographic and clinical characteristics of participants

Characteristics	Number (%)
Age, year, mean (SD), *n* = 207	64.35 (10.97)
BMI, kg/m^2^, mean (SD), *n* = 127	24.27 (16.57)
Cancer stage, *n* = 182	
I	9 (4.9)
II	35 (19.2)
III	39 (21.4)
IV	99 (54.4)
Total number of chemotherapy, mean (SD), *n* = 209	7.34 (6.0)
Sex, *n* = 209	
Male	114 (54.5)
Female	95 (45.5)
Comorbidities, *n* = 209	
Diabetes mellitus	86 (41.1)
Hypertension	88 (42.1)
Coronary artery disease	14 (6.7)
Chemotherapy regimen, *n* = 209	
5-fluorouracil	75 (35.9)
gemcitabine (Gemmis®)	67 (32.1)
nab paclitaxel (Abraxane®)	24 (11.5)
gemcitabine (Gemzar®)	22 (10.5)
fluoropyrimidine (TS-1®)	10 (4.8)
Oxaliplatin	3 (1.4)
Other	8 (3.8)

n, number; BMI, body mass index; SD, standard deviation; kg, kilogram; m, meter.

### Trajectory of physical performance

The hand grip strength and balance (as measured by the TUG test) remained stable over the chemotherapy treatment process. However, the aerobic capacity (2MST) changed significantly over time (*p* = 0.041) (Table 2). The Cochran’s Q test revealed that no significant change occurred in the proportion of participants who showed a positive Romberg test over time, χ^2^(4) = 4.333, *p* = 0.363.

**Table 2:** Physical function scores at different assessment time points

Variables	Baseline	2 months after 1st chemotherapy	3 months after 1st chemotherapy	4 months after 1st chemotherapy	6 months after 1st chemotherapy	*p*-value
Hand grip strength, kg, mean (SD)	26.97 (9.84)	25.20 (10.44)	25.69 (10.31)	24.83 (9.45)	25.15 (9.51)	0.390
TUG completion time, s, mean (SD)	13.30 (6.42)	14.45 (14.80)	13.15 (9.01)	12.59 (5.48)	12.44 (5.32)	0.198
2MST count, n, mean (SD)	57.82 (17.37)	54.18 (24.58)	55.69 (24.91)	54.34 (24.58)	51.95 (23.25)	0.041
Romberg test (positive), n (%)*	15 (7.2)	9 (4.3)	7 (3.3)	17 (8.1)	12 (5.7)	0.363

*Cochran’s Q test

kg, kilogram; TUG, timed up and go; 2MST, 2-minute step test; SD, standard deviation; n, number.

### Trajectory of CIPN symptoms

The CIPN scores measured by the EORTC QLQ-CIPN20 scale over time are shown in Table 3. When using ANOVA with repeated measures and Greenhouse-Geisser correction, the mean CIPN20 total scores were significantly different (F(3.080, 255.669) = 10.070, *p* < 0.001) at different follow-up times. Post hoc analysis with Bonferroni adjustment revealed that the CIPN20 total score significantly increased from pre-chemotherapy to 3-month follow-up (*p* = 0.026), from pre-chemotherapy to 4-month follow-up (*p* = 0.005), and from pre-chemotherapy to 6-month follow-up (*p* < 0.001). Significant changes in the CIPN20 total score were also found between the 2-month and 4-month follow-up and the 2-month and 6-month follow-up. The mean sensory, motor, and autonomic symptoms all significantly increased after chemotherapy.

**Table 3:** CIPN scores at different assessment time points

Variables	Baseline	2 months after 1st chemotherapy	3 months after 1st chemotherapy	4 months after 1st chemotherapy	6 months after 1st chemotherapy	*p*-value
Sensory, mean (SD)	3.03 (5.05)	5.40 (6.33)^a^	5.70 (6.62)^a^	6.12 (7.95)^a^	8.32 (10.44)^a^	< 0.001
Motor, mean (SD)	5.93 (7.70)	6.13 (7.77)	7.46 (9.12)	7.99 (9.48)^b^	9.19 (11.34)^a, b^	< 0.001
Autonomic, mean (SD)	12.05 (12.22)	13.56 (12.97)	16.26 (14.68)	14.93 (14.94)	16.28 (15.8)	0.017
Total, mean (SD)	21.01 (19.34)	25.09 (20.09)	29.41 (22.17)^a^	29.05 (24.02)^a, b^	33.79 (26.93)^a, b^	< 0.001

^a^ <0.05 compared to baseline

^b^ <0.05 compared to 2 months after the first chemotherapy

^c^ <0.05 compared to 3 months after the first chemotherapy

### Predictors of 6-month CIPN

The multiple linear regression analysis results showed that participants with a long TUG completion time before chemotherapy had a significantly higher CIPN20 total score (beta coefficient = 0.648, *p* = 0.003), sensory score (beta coefficient = 0.665, *p* = 0.011) and autonomic score (beta coefficient = 0.764, *p* = 0.002) at 6-month follow-up. A moderate, positive association between baseline proprioception (positive Romberg test) and the CIPN20 motor score (beta coefficient = 0.525, *p* = 0.009) at 6-month follow-up was also observed (Table 4).

**Table 4:** Outcomes of multiple linear regression analysis for association of 6-month CIPN with physical function at baseline (before chemotherapy), comorbidity, and CIPN at baseline

	CIPN– total (6-month)		CIPN– sensory (6-month)		CIPN– motor (6-month)		CIPN– autonomic (6-month)	
	Beta	95% CI	Beta	95% CI	Beta	95% CI	Beta	95% CI
Age	-0.148	-1.899 to 1.010	-0.537	-0.999 to 0.033	0.283	-0.391 to 1.255	-0.222	-1.308 to 0.521
BMI	0.180	-1.278 to 3.747	0.245	-0.387 to 1.395	0.240	-0.588 to 2.257	-0.026	-1.684 to 1.476
Cancer stage								
I	Reference		Reference		Reference		Reference	
II	-0.064	-60.174 to 50.553	-0.234	-24.851 to 14.411	0.212	-23.323 to 39.356	-0.173	-42.417 to 27.203
III	-0.199	-66.236 to 38.254	-0.493	-28.905 to 8.144	0.325	-17.975 to 41.174	-0.366	-48.060 to 17.639
IV	-0.029	-53.332 to 49.963	-0.305	-23.624 to 13.001	0.805	-5.460 to 53.012	-0.587	-52.622 to 12.324
Sex (male)	-0.031	-34.136 to 30.290	0.272	-6.459 to 16.384	-0.344	-28.881 to 7.588	0.105	-16.492 to 24.016
Diabetes mellitus	0.154	-16.190 to 33.975	0.246	-4.638 to 13.149	0.468	-0.474 to 27.923	-0.267	-24.858 to 6.683
Hypertension	-0.004	-23.159 to 22.746	-0.182	-11.348 to 4.929	0.105	-9.847 to 16.139	-0.004	-14.574 to 14.289
Coronary artery disease	0.047	-39.085 to 48.438	0.093	-12.745 to 18.288	-0.444	-47.291 to 2.253	0.414	-3.090 to 51.940
Hand grip strength, kg	0.088	-1.594 to 2.102	-0.166	-0.800 to 0.511	0.083	-0.925 to 1.168	0.162	-0.886 to 1.439
TUG completion time, s	0.684	1.374 to 5.518	0.665	0.266 to 1.735	0.069	-0.996 to 1.349	0.764	0.966 to 3.571
2MST count, n	-0.030	-0.637 to 0.542	-0.037	-0.226 to 0.192	-0.049	-0.373 to 0.294	0.011	-0.361 to 0.381
Romberg test (positive), n (%)	0.130	-20.750 to 46.889	-0.099	-14.949 to 9.035	0.525	7.501 to 45.790	-0.180	-31.883 to 10.645
CIPN-total (baseline)	0.186	-0.718 to 1.211	-0.137	-0.396 to 0.288	-0.563	-0.923 to 0.169	0.870	0.071 to 1.285
CIPN-sensory (baseline)	-0.033	-3.117 to 2.716	0.423	-0.475 to 2.004	0.091	-1.372 to 1.930	-0.524	-4.067 to 0.331
CIPN-motor (baseline)	0.387	-0.034 to 3.448	0.334	-0.346 to 1.228	0.227	-0.479 to 1.492	0.052	-1.259 to 1.532
CIPN-autonomic (baseline)	0.122	-0.718 to 1.211	-0.090	-0.396 to 0.288	-0.368	-0.923 to 0.169	0.569	0.071 to 1.285

BMI, body mass index; CIPN, chemotherapy-induced peripheral neurotoxicity; CI, confidence interval; kg, kilogram; TUG, timed up and go; 2MST, 2-minute step test; n, number; s, second.

## Discussion

In this secondary analysis of a longitudinal cohort study, the participants showed a significant decline in aerobic capacity and an increase in the severity of CIPN following chemotherapy treatment. Similar patterns were also observed for the sensory and motor neuropathy symptoms. The pre-chemotherapy TUG completion time and positive Romberg test were significantly associated with the CIPN20 score at 6-month follow-up. Overall, the results suggest that physical function, specifically mobility, balance, and proprioception, are predictive of the CIPN severity in individuals with pancreatic cancer scheduled for chemotherapy treatment.

This study found that 6 months after the commencement of chemotherapy, the severity of CIPN increased, while the aerobic capacity decreased. These findings are consistent with the study of Lønbro et al., who reported a lower VO_2_ peak during chemotherapy for a mixed cancer cohort [27], and that of Wang et al., who reported that the severity of CIPN increased gradually from pre-chemotherapy to completion of chemotherapy in newly-diagnosed breast cancer survivors [28]. Müller et al. also reported a significant deterioration of the CIPN signs/symptoms during chemotherapy for a mixed cancer cohort [29]. However, even though chemotherapy has been shown to reduce physical activity levels and physical fitness in cancer patients [30], it remains unclear whether the reduced aerobic capacity observed after chemotherapy is a result of chemotherapy-induced cardiovascular damage or cancer-related deconditioning. For example, it may be that the lower aerobic capacity observed in the present study is the result mainly of the advanced cancer stage of the participants. Previous studies showed that 40–60% of patients with metastatic gastrointestinal cancer experienced a substantial decline in physical function in the first 3 to 6 months of chemotherapy [31, 32]. Thus, future studies should investigate how chemotherapy affects activity levels and fitness in pancreatic cancer populations with varying cancer stages to better understand the relationship between physical activity levels and aerobic capacity during chemotherapy.

No significant differences were found in the hand grip strength, mobility, balance, and proprioception over time. These findings contrast with those of previous studies [29, 33]. For example, Monfort et al. reported significant negative changes in balance and walking speed after chemotherapy [34]. Morishita et al. similarly showed that cancer survivors had a lower balance function, as measured by the TUG time and area of the center of pressure, than healthy subjects [33]. Due to the limited evidence in the literature on physical function (e.g., grip strength, balance, and mobility) in patients with pancreatic cancer, more studies are required to confirm the results of the present study. In general, frailty has a significant impact on chemotherapy-related adverse outcomes in patients with gastrointestinal cancer [35] and on physical functions (including grip strength, gait speed, 6-minute walk test, Short Physical Performance Battery, and TUG test of elderly patients with cancer [36]. Thus, future studies could usefully examine the associations between frailty, physical function, chemotherapy-related adverse events (including CIPN), and mortality in patients with pancreatic cancer.

The present results showed that the baseline mobility and balance (as measured by the TUG test) were associated with an increased severity of CIPN six months after chemotherapy commencement. This finding is in line with previous studies that reported significant relationships between patient-reported CIPN and balance and walking [34, 37]. Significant positive correlations between CIPN severity and functional disability and balance deficits have also been reported [38]. The baseline balance/proprioception results obtained in the present study, as measured by the Romberg test, were significantly associated with the motor symptoms six months after the first chemotherapy session. Persistent motor neuropathy may have detrimental effects on physical function [39]. Therefore, future studies may also usefully examine the motor functions and fall risk in pancreatic cancer patients with CIPN.

While preventive interventions for CIPN have yet to be recommended [40], the preliminary evidence obtained in this study suggests that pre-chemotherapy physical function (i.e. mobility and balance) is associated with CIPN severity during chemotherapy. Therefore, pre-chemotherapy sensorimotor and balance training or physical therapy may be effective strategies in preventing or reducing CIPN severity [41]. A recent systematic review reported a limited number of studies supported an association between low physical activity and great CIPN risk in patients with breast, colorectal, ovarian, and mixed cancer types [42]. However, the review did not find any study that investigated physical activity levels before treatment [42]. The association between prechemotherapy physical function and the severity of CIPN delineates the link between levels of physical function, cardio-metabolic health, and susceptibility to comorbidities, such as diabetes and obesity - established risk factors of CIPN severity [13, 42]. Further research is required to elucidate the complex interplay between these factors and to evaluate the mechanisms and effectiveness of non-pharmacological physical therapy in preventing and treating CIPN in patients with pancreatic cancer [40].

### Study limitations

This study provides new insights into the trajectory and associations of physical function with CIPN symptom severity in patients with pancreatic cancer. However, several limitations should be noted. First, since 75% of the participants had advanced (stage III or stage IV) pancreatic cancer, the findings may not be generalizable to all pancreatic cancer populations. Moreover, the present sample size only allowed a limited number of potential factors to be included in the regression models. For example, factors such as the type of medication, the route of medication delivery, and the chemotherapy dosage, may all affect the development of CIPN [43]. Future studies should thus assess and report the chemotherapeutic regimens for pancreatic cancer more clearly. Missing data in some demographic and clinical characteristics (i.e., age, BMI, and cancer stages) were due to the participants failing to respond to the questionnaire, incomplete medical records, and safety reasons, and may lead to biased results. Finally, there is currently no gold standard assessment tool for CIPN [44]. The present study employed the CIPN20 patient self-reported questionnaire to assess the participants’ experience of symptoms and functional limitations related to CIPN [45]. However, future studies should consider including clinician-based assessments for CIPN in addition to patient-reported outcome measures [46].

## Conclusions

The findings of this study indicate that the TUG completion time and a positive Romberg test before chemotherapy are both predictive factors of the severity of CIPN 6 months after the commencement of chemotherapy. This finding suggests that the TUG time and Romberg test may be important functional tests for patients with pancreatic cancer. Therefore, healthcare professionals working with patients with pancreatic cancer should consider encouraging or offering exercise as a possible approach for preventing or managing the CIPN severity in patients who are scheduled for chemotherapy. Further studies with a larger sample size [47] are required to confirm the present findings and better understand the effects of pre-chemotherapy physical function on changes in the occurrence and severity of CIPN over the course of chemotherapy treatment.

## Data Availability

The datasets used and/or analyzed during the current study available from the corresponding author on reasonable request.
